# Comparing perioperative vaginal misoprostol with intraoperative pericervical hemostatic tourniquet in reducing blood loss during abdominal myomectomy: A randomized controlled trial

**DOI:** 10.4274/jtgga.galenos.2018.2018.0049

**Published:** 2019-02-26

**Authors:** Muhibat A. Afolabi, Grace G. Ezeoke, Rakiya Saidu, Munirdeen A. Ijaiya, Abiodun S. Adeniran

**Affiliations:** 1 Department of Obstetrics and Gynecology, University of Ilorin Teaching Hospital, Ilorin, Nigeria; 2 Department of Obstetrics and Gynecology, University of Ilorin, Ilorin, Nigeria

**Keywords:** Uterine leiomyoma, misoprostol, hemostatic tourniquet, abdominal myomectomy, hemostasis

## Abstract

**Objective:**

To compare the effectiveness of perioperative vaginal misoprostol with intraoperative pericervical hemostatic tourniquet in reducing blood loss during abdominal myomectomy.

**Material and Methods:**

A randomized controlled trial involving women with uterine leiomyoma who underwent abdominal myomectomy was conducted at a tertiary facility in Nigeria. Participants were recruited after they gave informed consent and randomized into group I (single dose 400 μg vaginal misoprostol one-hour before surgery) and group II (intraoperative pericervical hemostatic tourniquet). Eighty participants (40 in each group) were recruited. Uterine size was measured in centimeters above the pubic symphysis, and blood loss estimation involved direct volume measurement and gravimetric methods. The main outcome measures were intraoperative blood loss, blood transfusion, and recourse to hysterectomy. Ethical approval and trial registration were obtained; the data were analyzed using the SPSS software version 21.0; p<0.05 was considered significant.

**Results:**

Participants in group I had higher mean intraoperative blood loss (931.89±602.13 vs 848.40±588.85 mL, p=0.532), intra-operative blood transfusion rates (60 vs 55%; p=0.651) and mean units of blood transfused (1.30±1.20 vs 1.20±1.30; p=0.722) compared with group II. The mean uterine size (19.50±6.93 vs 20.05±6.98 cm; p=0.725) and number of fibroid nodules (11.25±7.99 vs 11.45±8.22; p=0.912) were comparable. The change in post-operative hematocrit was 2.66±2.21% vs 3.24±2.85% (p=0.315) and post-operation blood transfusion was 2.5 vs 5% (p=0.556). There was no recourse to hysterectomy in either of the study groups. While adverse effects of misoprostol occurred in 5 (12.5%) participants of group I.

**Conclusion:**

The effectiveness of perioperative vaginal misoprostol is comparable to intra-operative hemostatic pericervical tourniquet in reducing blood loss during abdominal myomectomy.

## Introduction

Uterine leiomyoma is the most common benign genital tract tumor in women of reproductive age (1) and the most frequent reason for gynecologic consultation in most Nigerian hospitals (2,3). It is more common among black women (4) with an incidence of 3.0% to 29.3% (2,5). It may be asymptomatic with incidental discovery during pelvic examination or ultrasonography for other indications. Symptomatic uterine fibroid can adversely affect the quality of life, especially in low resource countries where patients often present late with huge masses and anemia (2,3,5). The definitive treatment for symptomatic leiomyoma is hysterectomy; however, for women who desire future fertility or preservation of the uterus, myomectomy is a common option (6). Abdominal myomectomy is often preferred to the laparoscopic route in the presence of large and multiple uterine leiomyoma (7). 

Intraoperative hemorrhage necessitating blood transfusion is the most common complication of abdominal myomectomy (4) and when uncontrollable, it may necessitate hysterectomy (8). The volume of blood loss at abdominal myomectomy depends on the uterine size, number, and location of the leiomyoma (7). Generally, the blood transfusion rate for abdominal myomectomy is 13.5 to 58.2% (4,9), with a 2% inadvertent hysterectomy rate following uncontrollable hemorrhage (6,9). Therefore, effective interventions to reduce blood loss remain desirable during myomectomy (10). 

Methods of reducing hemorrhage during myomectomy include peri-operative vaginal misoprostol, intra-operative-myometrial vasopressin, intra-myometrial bupivacaine with epinephrine, intravenous tranexamic acid, gelatin-thrombin matrix, intravenous ascorbic acid, vaginal dinoprostone, loop ligation of the leiomyoma pseudo-capsule, fibrin sealant patch and per cervical tourniquet using a Foley catheter (3). However, each method has its limitations; therefore, controlling hemorrhage remains a major task for gynecologists (11). 

The Foley catheter, often improvised as a tourniquet in low resource countries, is cheap and readily available (12); however, they require intermittent release intraoperatively to prevent the build-up of toxins and tissue ischemia, and may be impracticable to apply sometimes (11). Once the tourniquet is removed, there is bleeding from the raw myometrium and cavities with the possibility of increased blood loss and blood transfusion (13).

Misoprostol, in addition to its role in managing miscarriages, pre-induction cervical ripening, and induction of labor, prevention and treatment of primary postpartum hemorrhage has gained relevance in myomectomy (14). It increases myometrial contractions thereby reducing uterine artery blood flow to the uterus (15). Its heat stability and long shelf-life improves its availability in the tropics where huge leiomyoma are predominant, and the multiple routes of administration increases choices and acceptability (16). The peak plasma level of 400 μg of vaginal misoprostol is reached one to two hours after administration and is sustained for four hours, and the side effects are self-limiting (15,16). 

Reports on misoprostol use to control blood loss in abdominal myomectomy showed a reduction in intra and post-operative blood loss, surgical time, and post-operative blood transfusion (6,15), but these were from developed countries. In practice, there are instances when application of the tourniquet is impracticable and another method is indicated. Therefore, aim of this study was to evaluate the effectiveness of vaginal misoprostol compared with hemostatic tourniquet in reducing hemorrhage during abdominal myomectomy.

## Material and Methods

### Study design

The study was a randomized controlled study conducted between June 2016 and May 2017 at the University of Ilorin Teaching Hospital - a tertiary health facility in North-central Nigeria with facilities for undergraduate and postgraduate medical training. The obstetrics and gynecology department has 170 beds, an average annual delivery rate of 2000 and 500 gynecologic admissions. Participants were women with symptomatic uterine leiomyoma who underwent abdominal myomectomy. The study evaluated the effectiveness of perioperative misoprostol administered vaginally compared with intraoperative pericervical hemostatic tourniquet in reducing blood loss during abdominal myomectomy. 

The inclusion criteria were a diagnosis of uterine leiomyoma and a decision for abdominal myomectomy. Women who had other forms of myomectomy, allergy to prostaglandins, chronic medical disorders, previous uterine surgery (myomectomy, caesarean delivery) and anemia (hematocrit <10 g/dL at 24 hours preop) were excluded from the study. The primary outcome measures were estimated intraoperative blood loss and the need for intraoperative blood transfusion. The secondary outcome measures were intraoperative recourse to hysterectomy, post-operative hematocrit change, and adverse effects of misoprostol among group I participants.

### Sample size determination

The sample size was calculated using a previously validated formula (17). The power was set at 95%. The standard normal deviate corresponding to 5% level of significance and the mean intraoperative blood loss for hemostatic tourniquet (18) and misoprostol (15) of 286.4±137.5 mL and 200.16±18.8 mL from previous studies with 10% attrition rate yielded a sample size of 40 participants for each group and a total 80 participants.

### Study protocol

All women with symptomatic uterine leiomyoma were informed about the study; interested individuals were then screened using the eligibility criteria and eligible women were requested to provide written informed consent. Consenting participants were randomized into one of two groups to receive perioperative vaginal misoprostol (group I) or intraoperative pericervical hemostatic tourniquet (group II). To allow randomization, researchers prepared the management protocol for each group and sealed one protocol per envelope with a computer-generated number assigned. Randomization was performed by picking the numbered study envelopes sequentially and managing the participant based on the enclosed protocol. Participants were identified with the randomization number until discharge from the hospital. All participants had standard preoperative evaluations including complete blood count; urinalysis; serum electrolyte; urea and creatinine; pelvic ultrasound scan for size, number and location of the leiomyoma; and hysterosalpingography. Other investigations were performed as indicated. 

Group I participants received two tablets of 200 μg i.e. total of 400 μg misoprostol (Pfizer Limited, United Kingdom) administered into the posterior fornix of the vagina at least one hour before the onset of surgery. Group II participants had peri-cervical tourniquet using a Foley catheter size 18, which was firmly tied at the level of the cervico-isthmic junction of the uterus before the uterine incision. The time of tourniquet administration was recorded, and it was released not later than 45 minutes after its application. For those requiring multiple applications, the tourniquet was reapplied after a period of at least 15 minutes. 

All procedures were performed in equal proportion by four consultant gynecologists of similar skill and experience, and estimation of blood loss was performed through the measurement of blood volume in the suction bottle; other losses were accounted for using gravimetric methods by mopping with pre-weighed abdominal mops and repeat measurements with a one-gram weight difference equivalent to 1 mL of blood (19). The maximum allowable blood loss (transfusion trigger) was calculated for each participant and intraoperative blood transfusion was commenced when this was exceeded (20). Blood transfusion was also commenced with cardiovascular instability from hemorrhage or signs of inadequate perfusion or oxygenation. All participants were monitored until hospital discharge. Participants in group I were evaluated for adverse effects of misoprostol [nausea, vomiting, diarrhea, elevated temperature (>38 °C), shivering] within one hour and 24 hours post-surgery. All participants had a hematocrit estimation at 24 hours post-surgery in addition to other routine post operation care procedures.

### Ethical issues

Ethical approval was obtained from the ethical review committee of the University of Ilorin Teaching Hospital, Ilorin, Nigeria (ERCPAN/2015/09/1455; 10/09/2015) before commencement of the study. The trial was registered with the Pan African Clinical Trial Registry (www.pactr.org) with registration number PACTR201802003039106. Written informed consent was obtained from all participants in the study.

### Statistical analysis

The data obtained from this study were analyzed using SPSS version 21.0. The chi-square, t-test, and Mann-Whitney U test were used to describe variables as appropriate. P<0.05 was considered significant.

## Results

**Figure 1:**
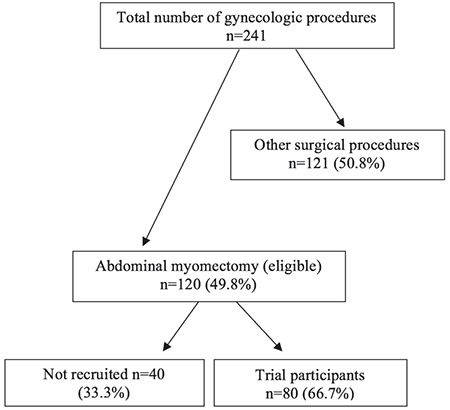
Flow chart of participants in the trial

A total of 120 women were screened for eligibility in the study. Eighty (66.7% of the total screened) women participated in the study, 40 in each group. Figure 1 shows the flow chart for the trial. The mean age of the participants was 36.4±5.97 (range, 24-45) years; 47 (58.8%) were nulliparous, and 51 (63.8%) had no children. The common presenting symptoms were excessive/prolonged menstrual flow (n=50; 62.5%), abdominal swelling (n=46; 57.5%), inability to conceive (n=43; 53.75%), and 75 (93.7%) had multiple symptoms (Table 1). 

There were similarities in the two groups in relation to mean uterine size (19.50±6.93 vs 20.05±6.98; p=0.725) and mean number of fibroid nodules (11.25±7.99 vs 11.45±8.22; p=0.912), as shown in Table 2. Intraoperatively, group I participants recorded higher values in mean intraoperative blood loss (931.89±602.13 vs 848.40±588.85 mL; p=0.532), blood transfusion [24 (60.0%) vs 22 (55.0%), p=0.651], and mean number of units of blood transfused (1.30±1.20 vs 1.20±1.30, p=0.655) than group II. Postoperatively, blood transfusion was performed in 1 (2.5%) patient and 2 (5.0%) patients in groups I and II, respectively, as shown in Table 3. 

The mean preoperative hematocrit was 33.78±2.70 vs 34.56±0.53% (p=0.253), the mean post-operative hematocrit was 31.11±3.25 vs 31.32±3.36% (p=0.782), and the mean hematocrit change was 2.66±2.21 vs 3.24±2.85% (p=0.315) for groups I and II, respectively. Among the 34 participants [group I (n=16) and group II (n=18)] who did not receive intraoperative blood transfusion, the hematocrit change was similar ranging from 2 to 10.5%, but these were not statistically significant (Table 4). Adverse effects of misoprostol occurred in 5 (12.5%) patients of group I, II (40.0%) of whom experienced multiple adverse effects (Table 5).

## Discussion

In this study, the mean intra-operative blood loss, rate of intra-operative blood transfusion, and number of units of blood transfused were higher among participants who had perioperative vaginal misoprostol compared with those who had intra-operative pericervical hemostatic tourniquet, although these were not statistically significant. However, the change in post-operative hematocrit and rate of post-operation blood transfusion were higher in women who had intra-operative pericervical hemostatic tourniquet, but again, these were not statistically significant. None of the participants in the study had recourse to hysterectomy, and the adverse effects of misoprostol were minimal and self-limiting. 

The strength of the study is that it compared perioperative misoprostol with hemostatic tourniquet, which is uncommon in the literature, the randomized design, and the objective measurement of blood loss. The limitations included the small sample size and the limited study area. 

Misoprostol has been reported to be effective in reducing blood loss in myomectomy following comparison with placebo (21,22,23) or other agents (24). The mean blood loss following misoprostol administration in this study was higher than in reports from Egypt (574±194.8 mL) (23), Iran (458±287 mL) (22), and Turkey (472±77 mL) (21). A review of the methodology showed that these studies with lower blood loss had lower mean uterine sizes of <24 weeks (23), 8.7±4.6 weeks (22), and 15.7±2.6 weeks (21) compared with 19.50±6.93 in this study. This may explain the higher blood loss in our study because blood loss during myomectomy has been shown to be proportional to uterine size. In addition, the number of fibroid nodules is related to blood loss, which explains the lower blood loss in the Turkish study with mean number of leiomyoma of 5.5±1 (21) compared with 11.25±7.99 in this study. Blood loss estimation remains central during abdominal myomectomy. In the Turkish (21) and Iranian (22) studies, the recorded blood loss was measured from the blood in the suction bottle, whereas this study assessed other losses in addition to the suction bottle, using a gravimetric method. 

Different studies used different dosing regimens for misoprostol in myomectomy. A study from Iran used 200 mg of vaginally administered misoprostol three hours prior to surgery (22) compared with 400 μg of vaginal misoprostol administered one hour prior to surgery in this study. This raises a future research question on the effect of dose and time interval from administration of misoprostol to commencement of surgery on blood loss. Another study evaluated the effect of multiple doses and reported greater blood loss (200.16±18.8 vs 101.4±25.5 mL) (15) for single and two doses, respectively, but more adverse effects of misoprostol with multiple dosing. The concern of researchers remains the possible additive adverse effects of misoprostol with multiple dosing (15), which necessitated single dosing in this study. However, further comparison was limited by the smaller mean uterine size and number of leiomyomas (15.33±8.48 weeks and 2.91±4.24 leiomyomas) in a previous study (15) compared with this study.

The intra-operative blood transfusion rate of 60% in this study was higher than in previous reports with 15.3% (21) and 24% (23); some studies reported no blood transfusion requirement (15,22). This may be attributed to the larger uterine sizes, presence of multiple uterine fibroids, and greater mean blood loss in this study. There is no consensus on the time to initiate blood transfusion during myomectomy. In this study, intraoperative blood transfusion was commenced when the calculated maximum allowed blood loss for the patient was reached or evidence of cardiovascular instability from hemorrhage. However, another study employed a loss of 2000 mL as indication for blood transfusion (22). 

In this study, the mean pre-operative and post-operative hematocrits and changes in hematocrit were not statistically significant. This emphasizes the comparative effectiveness in reducing blood loss by the two methods compared. In most studies that compared misoprostol with placebo, the misoprostol group had post-operative hemoglobin values that were significantly higher than those found in the placebo group (21,22,23). This validated the effectiveness and superiority of misoprostol, which is an active agent over placebo in such studies.

The adverse effects of misoprostol experienced by participants in this study compared to those in previous reports; they were self-limiting and included nausea, vomiting, diarrhea, shivering, and fever (15,21,22,23). They either required no intervention or simple interventions to alleviate symptoms such as covering the patient with blankets for shivering.

Other researchers compared misoprostol with other agents in reducing blood loss in myomectomy. A comparison of the effectiveness of the administration of a single preoperative dose of vaginal misoprostol with intraoperative oxytocin infusion on blood loss during abdominal myomectomy reported statistically significantly lower blood loss (401±48 vs 589±49 mL) in the misoprostol group (24). Also, a study that compared rectal misoprostol plus perivascular vasopressin with perivascular vasopressin alone reported statistically significantly lower blood loss (334 vs 623 mL) in the former group (25). This implies that misoprostol compares favorably with other agents, depending on the locally available alternatives in a number of settings. 

The estimated blood loss from the tourniquet group in this study was higher than that reported by Ikechebelu et al. (11) in Nnewi, Nigeria (515.7±292.81 mL). A possible explanation is the difference in blood loss estimation in the studies. A comparison of tourniquet and no-tourniquet use recorded a significant reduction in blood loss in the tourniquet group (11,18), yet tourniquet use produced greater blood loss when compared with other hemostatic techniques such as vasopressin or preliminary uterine artery ligation (25,26). However, there is paucity of data regarding comparisons of tourniquet use and misoprostol in reducing blood loss during abdominal myomectomy.

In summary, this study suggests comparable effectiveness in reducing blood loss during abdominal myomectomy for peri-operative vaginal misoprostol and intra-operative hemostatic peri-cervical tourniquet. This is recommended for routine use especially in instances where application of the tourniquet is impracticable due to significant pelvic adhesions and leiomyoma in the broad ligament, uterine isthmus or cervix.

## Figures and Tables

**Table 1:**
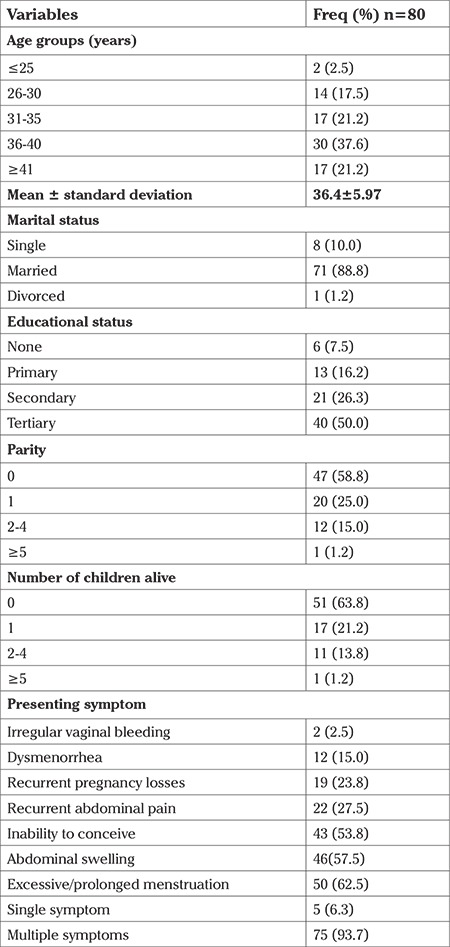
Sociodemographic characteristics of the study population

**Table 2:**
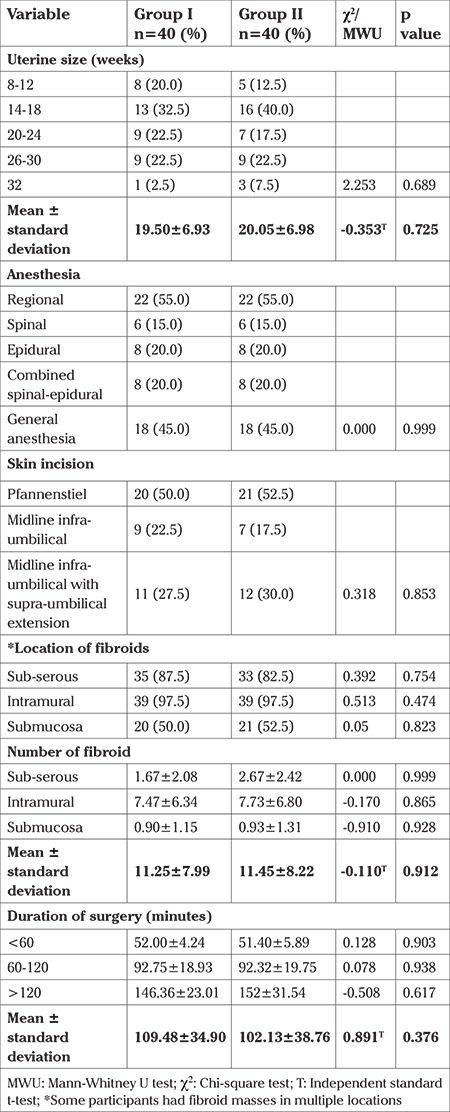
Intra-operative events of participants in the two groups

**Table 3:**
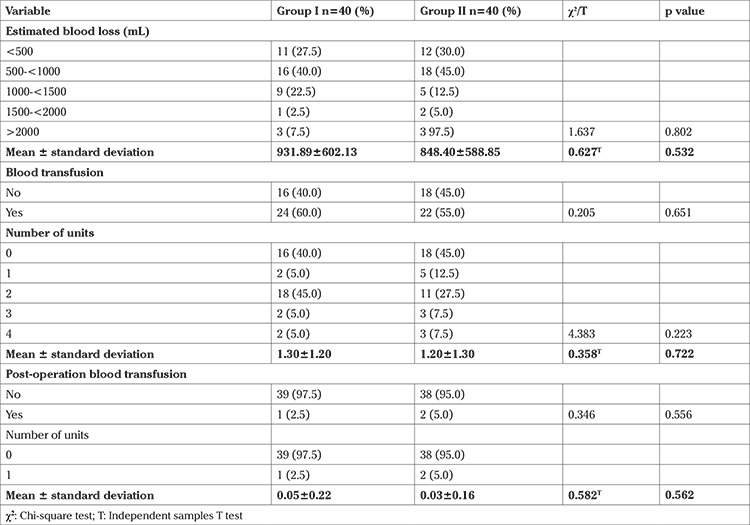
Pre-operative, intra-operative, and post-operative intravenous fluid and blood transfusion management

**Table 4:**
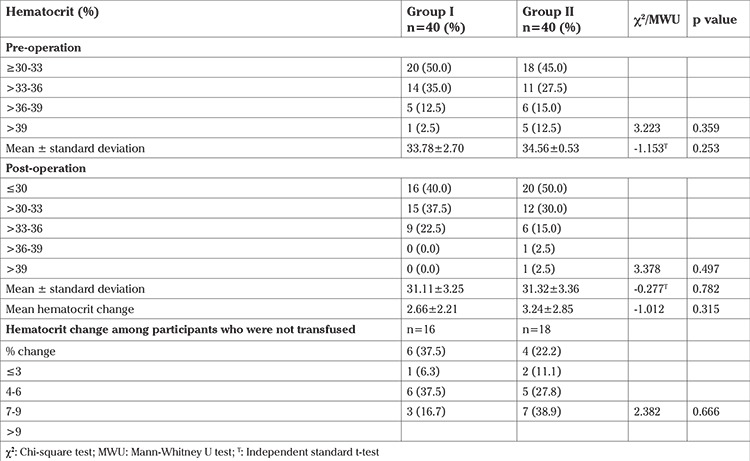
Comparison of levels and changes in hematocrit among participants

**Table 5:**
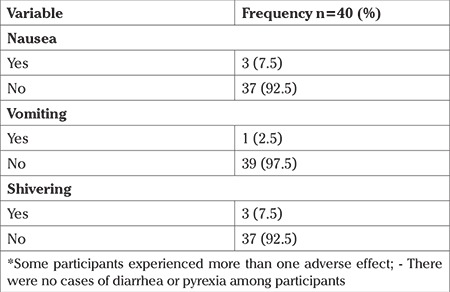
Adverse effect profile of misoprostol among group I participants*
